# Studies on the alleviating effect of *Bifidobacterium lactis* V9 on dextran sodium sulfate-induced colitis in mice

**DOI:** 10.3389/fmed.2025.1496023

**Published:** 2025-01-24

**Authors:** Xiaoyan Duan, Rilige Wu, Jianbo Li, Zeya Li, Yanqi Liu, Ping Chen, Bangmao Wang

**Affiliations:** ^1^Department of Gastroenterology, General Hospital, Tianjin Medical University, Tianjin, China; ^2^Tianjin Institute of Digestive Diseases, Tianjin, China; ^3^Tianjin Key Laboratory of Digestive Diseases, Tianjin, China; ^4^Department of Gastroenterology, Affiliated Hospital of Inner Mongolia Medical University, Hohhot, China; ^5^Department of Nuclear Medicine, Affiliated Hospital of Inner Mongolia Medical University, Hohhot, China; ^6^Inner Mongolia Key Laboratory of Molecular Imaging, Hohhot, China

**Keywords:** *Bifidobacterium lactis* V9, ulcerative colitis, intestinal barrier, intestinal flora, tryptophan metabolism

## Abstract

**Background:**

Inflammatory bowel disease (IBD) has become a global public health problem with complex pathogenesis and limited therapeutic options. We aimed to investigate the potential mechanisms by which *Bifidobacterium lactis* V9 (V9) alleviated colitis in a dextran sodium sulfate-induced colitis model mice.

**Methods:**

Mice were induced to develop colitis by drinking DSS solution to induce colitis. The expression of the relevant factors in the blood supernatant of the mice was determined by ELISA. RT-qPCR and Western blotting were used to detect mRNA and protein expression of target genes. The fecal microbiota was analyzed by 16S rRNA sequencing. Intestinal metabolites were analyzed by untargeted metabolomics;

**Results:**

V9 effectively improved the overall symptoms of the colitis model mice. H&E showed that V9 re-stored the intestinal tissue structure. ELISA showed that V9 decreased the levels of IL-6, IL-22, and TNF-α and increased IL-10, SP, VIP, and 5-HT. V9 increased the expression of AHR, CYP1A1, MUC2, Claudin-3, Occludin, and ZO-1, and decreased 5-hydroxytryptamine transporter and Claudin-2. V9 increased the abundance of gut microbiota in colitis mice to promote the growth of beneficial bacteria. V9 increased tryptophan metabolites, and short-chain fatty acids, and improved gut inflammation.

**Conclusion:**

V9 attenuates intestinal inflammation, improves the mucosal barrier, modulates intestinal microecology and exerts a protective effect in a mouse model of DSS-induced colitis.

## 1 Introduction

Ulcerative colitis (UC) is a subtype of inflammatory bowel disease (IBD), a chronic, non-specific inflammatory bowel disease. Clinical symptoms include diarrhea, blood in the stool, abdominal pain and even systemic symptoms such as fever, weight loss and fatigue. The pathogenesis of IBD is complex and its development is influenced by a number of factors including genetic susceptibility, immune regulation, intestinal barrier integrity and intestinal flora (1). Intestinal flora is significantly correlated with IBD, and dysbiosis may induce the development of IBD or be one of the pathological signs of IBD (2).

Traditional drugs used clinically to treat UC include 5-aminosalicylic acid drugs, glucocorticoids, immunosuppressants, biologics and other newer drugs, but these drugs suffer from high side effects, high drug resistance, high costs and poor patient compliance. As a result, micro-ecological agents have attracted much attention (3), and several studies have shown that probiotics are effective in improving the symptoms of UC, acting by regulating the balance of intestinal flora, improving the function of the intestinal mucosal barrier, and inhibiting the growth of harmful microorganisms, thereby improving the symptoms of intestinal inflammation in patients (4). Guidelines for the treatment of UC with probiotics have been recommended (5).

*Bifidobacterium lactis* V9 (V9) is a probiotic strain isolated from the intestinal tract of healthy Mongolian children in Inner Mongolia in 2005 and identified by 16S rDNA as *Bifidobacterium animalis* subsp. lactis (6). V9 has a strong tolerance to artificial gastric and intestinal fluids, with a survival rate of 99.7% after anaerobic incubation in artificial gastric fluid at pH 2.0 for 3 h followed by access to artificial intestinal fluid at pH 8.0 for 8 h of digestion, and it can tolerate 0.3% bovine bile salts (6), therefore V9 has a stable colonization ability in the intestinal tract of patients (7). V9 has been shown to have positive therapeutic potential in a variety of diseases (7, 8), including irritable bowel syndrome (9) and diarrhea (10).

A previous study found that oral administration of a probiotic complex including V9 was effective in relieving symptoms in patients with UC (11), but whether oral ad-ministration of V9 alone can have a similar effect has not been reported. Therefore, a single strain of V9 was selected for this study. The aim is to determine that V9 has an alleviating effect on the symptoms of dextran sulfate sodium (DSS)-induced colitis model mice, and that the intervention of V9 can alter the expression of serum inflammatory factors, neurotransmitters and intestinal barrier proteins and other related factors, and have an effect on the composition and metabolites of the intestinal microbiome, so as to provide more evidence for the alleviation of colitis by V9, and to offer new insights into the intestinal microecological therapy for colitis.

## 2 Materials and methods

### 2.1 General

C57BL/6 J mice were provided by Beijing Vital River Laboratory Animal Technology Co., Ltd. V9 was provided by the Key Laboratory of Dairy Biotechnology and Engineering, Ministry of Education, Inner Mongolia Agricultural University, China. DSS (36,000–50,000 Da) was purchased from MP Biomedicals (Santa Ana, CA, United States). ELISA kits were purchased from Wuhan ELISA Lab Biotech Co., Ltd. (China). RNA isolater Total RNA Extraction Reagent and HiScript II Q RT SuperMix for qPCR (+gDNA wiper) kit were purchased from Nanjing Vazyme Biotech Co., Ltd. (China). Primers were purchased from Beijing Tsingke Biotech Co. (China). Protease inhibitors were purchased from Roche® Life Science (F. Hoffmann-La Roche Ltd., Basel, Switzerland). SDS-PAGE gel kit, BCA protein assay kit and RIPA lysate were purchased from Beijing Solarbio Science & Technology Co. (China). ECL chemiluminescence kit was purchased from Beyotime Biotech Inc. (Shanghai, CHINA). Methanol, acetonitrile and formic acid were purchased from Thermo Fisher Scientific Inc. (Waltham, MA, United States). Acetic acid was purchased from Sigma-Aldrich LLC. (Merck KGaA, Darmstadt, Germany). All other reagents were purchased from Sinopharm Chemical Reagent Co. (Shanghai, China) unless otherwise stated.

### 2.2 Establishment and treatment of colitis model mice

C57BL/6 J mice (male, 6–8 weeks, 20–22 g) were housed in an SPF laboratory (22 ± 2°C, 55 ± 10% humidity, 12 h light/dark cycle) and fed an ad libitum with SPF mouse chow and sterilized water.

**Figure 1:**
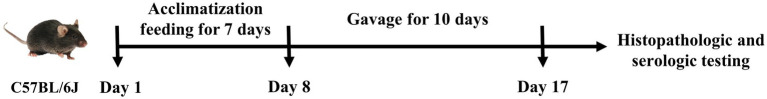
Schematic of the experimental study of V9 intervention in DSS-induced colitis in mice.

After 7 days of acclimatization, the mice were randomly divided into three groups (*n* = 6 in each group): control group (group C), model group (group M) and group V9. The experimental procedure was as shown in Figure 1 and Table 1. From day 8, mice in group C were given water ad libitum; mice in groups M and V9 were given DSS solution (1.5% dissolved in water) ad libitum to induce colitis. From day 8 to day 17, mice in groups C and M were given saline by gavage, while mice in group V9 were given V9 solution (4 × 10^9^ CFU, resuspended in saline) by gavage.

**Table 1:** Subgroups and intervention protocols in V9 intervention study in the DSS-induced colitis in mice.

Groups	Number	Drinking	Drugs	Administration	Frequency	Duration
C	*n* = 6	Water	Saline	Gavage	1 time/day	10 days
M	*n* = 6	1.5% DSS	Saline	Gavage	1 time/day	10 days
V9	*n* = 6	1.5% DSS	V9 (4.0 × 10^9^ CFU)	Gavage	1 time/day	10 days

The protocol of this study was approved by the Medical Ethics Committee of Inner Mongolia Medical University (YKD202302004). All animal experiments were per-formed in accordance with the National Institutes of Health guidelines for the care and use of laboratory animals.

### 2.3 Measurement of various parameters in experimental mice after V9 intervention

Disease Activity Index score (DAI score): used to assess the severity of colitis, i.e., the sum of body mass, fecal consistency and fecal occult blood (12). Calculation formula: DAI = (weight loss score + stool consistency score + fecal occult blood score) ÷ 3.

Measurement of colon length: After the intervention in each group of mice ac-cording to the experimental protocol, the mice were fasted without food and water for 24 h. After the mice were killed by cervical dislocation method, the abdomen was dissected and the colon was removed and its length measured.

Pathological analysis: 1 cm of distal colon tissue was removed, fixed in 4% para-formaldehyde, dehydrated, paraffin-embedded, sectioned, stained, and then microscopically examined, and images of colon tissue sections were evaluated using Slide Viewer 2.5 software (3DHISTECH Ltd., Budapest, Hungary). Histological grading criteria (13) were based on four areas: (1) loss of intestinal epithelial cells, (2) proliferation of connective tissue, (3) infiltration of inflammatory cells, and (4) cellular oedema.

### 2.4 Determination of serum cytokines

After the mice in each group were treated according to the experimental protocol, blood was collected through the orbital venous plexus, allowed to stand at room temperature for 20 min, then centrifuged at 4,000 rpm for 20 min at 4°C, and the blood supernatant was transferred to EP tubes and stored at −80°C in the refrigerator.

The experiments were performed according to the procedure of the ELISA kit instructions for the determination of serum concentrations of interleukin (IL)-6, IL-10, IL-22, tumor necrosis factor (TNF)-α, substance P (SP), vasoactive intestinal peptide (VIP) and 5-serotonin (5-HT) and other factors.

### 2.5 Quantitative reverse transcription PCR

Total RNA was extracted from colon tissue stored at-80°C using the TRIzol method. The OD260, OD280 and OD260/OD280 values were measured using a NanoVue Plus spectrophotometer (GE HealthCare., California, United States) to determine the purity and concentration of the RNA. Experiments were performed according to the HiScript II Q RT SuperMix for qPCR (+gDNA wiper) kit instructions to determine the expression level of the target genes, and the relative expression values of the target genes were calculated using the 2^−ΔΔCt^ method. Table 2 shows the primer sequences for target genes, with Mus GAPDH as the internal reference gene.

**Table 2:** Primer sequences for target genes.

Gene	Primer	Sequence (5′-3′)	PCR products
Mus GAPDH	Forward	ATGGGTGTGAACCACGAGA	229 bp
	Reverse	CAGGGATGATGTTCTGGGCA	
Mus AHR	Forward	CGAGATCTCCAGCCCTTTCT	194 bp
	Reverse	CTAACAGCGCAGGGCTTGAA	
Mus SERT	Forward	GTGAACTGCATGACGAGCTT	216 bp
	Reverse	ACTATCCAAACCCAGCGTGA	
Mus CYP1A1	Forward	CTTCCGGCATTCATCCTTCG	239 bp
	Reverse	GCTTGCCCAAACCAAAGAGA	
Mus MUC2	Forward	TGACAATGTGCCCAGAGAGT	223 bp
	Reverse	AGCTTTGCATCGTTTGGTGT	
Mus Claudin 2	Forward	TTAGGACTTCCTGCTGACAT	149 bp
	Reverse	ACTACAGCCACTCTGTCCTT	
Mus Claudin 3	Forward	AGATGGGAGCTGGGTTGTAC	182 bp
	Reverse	GTAGTCCTTGCGGTCGTAGG	
Mus Occludin	Forward	ACAGTCCAATGGCCTACTCC	162 bp
	Reverse	TACCATTGCTGCTGTACCGA	
Mus ZO-1	Forward	CCAGCAACTTTCAGACCACC	154 bp
	Reverse	TTGTGTACGGCTTTGGTGTG	

### 2.6 Western blotting

Colon tissues stored at −80°C in a refrigerator were ground in liquid nitrogen, RIPA lysate and protease inhibitor were added, and total proteins were extracted by centrifugation. Experiments were performed according to the instructions of the BCA Protein Concentration Measurement Kit and the concentration of target proteins was determined. Protein bands were separated by 10% SDS-PAGE, then transferred to PVDF membrane and blocked with 5% skimmed milk powder for 1 h. Primary anti-body was incubated overnight at 4°C, secondary antibody was incubated for 1 h at room temperature and color developed using ECL chemiluminescence kit. Protein bands were analyzed using AlphaEaseFC (Alpha Innotech, San Leandro, California, United States).

Primary antibodies against AHR, CYP1A1 were purchased from Santa Cruz Bio-technology, Inc. (Heidelberg, Germany). Primary antibodies against SERT and Clau-din-3 were purchased from Abways Technology, Inc. (Shanghai, China). Primary anti-body against MUC2 was purchased from Abcam Limited (Shanghai, China). Primary antibodies against Claudin-2, Occludin and ZO-1 were purchased from Proteintech Group, Inc. (Wuhan, China). GAPDH was used as an internal reference protein, and its primary antibody and secondary antibodies for all target proteins were purchased from Wuhan Servicebio Technology Co., Ltd. (Wuhan, China).

### 2.7 16S rRNA sequencing

Samples of cecal contents from each group were collected from the refrigerator at −80°C. Total genomic DNA from fecal bacteria was extracted according to the instructions of the QIAamp Fast DNA Stool Mini Kit (Germantown, MD, United States). Primers were designed to match the V3-V4 variable region of the 16S rRNA gene, PCR amplification was performed and the PCR products were electrophoresed on a 2% agarose gel and purified, re-quantified and homogenized to generate a sequencing library using the Qiagen Gel Extraction Kit (Qiagen, Germany). QC-qualified libraries were sequenced on NovaSeq 6000 (Illumina, Inc., San Diego, CA, United States) and OTUs/ASV analysis was performed using BMKCloud (www.biocloud.net). USEARCH software was used to cluster PCR products to analyze the diversity and composition of the gut microbiota. Representative sequences of OTUs were classified into species using the Ribosomal Database Project (RDP) classifier. Alpha and beta diversity indices were evaluated using QIIME 2 software to calculate the relative abundance of gut microorganisms and expressed as percentages.

### 2.8 Metabolomic analysis of cecal contents

Samples were thawed at room temperature, 100 mg of cecal content sample was weighed, 400 μL of pre-cooled methanol/acetonitrile/water solution (4:4:2, v/v) was added, mixed by vortexing and allowed to stand at −20°C for 1 h. The sample was centrifuged at 14,000 g for 20 min at 4°C. The supernatant was lyophilized under vacuum and stored at −80°C in a refrigerator. The sample was reconstituted with 100 μL aqueous acetonitrile (1:1, v/v), centrifuged at 14,000 g for 15 min at 4°C and 2 μL of the supernatant was injected into the sample for analysis.

The chromatographic separation of the samples was performed on an ultra-high pressure liquid chromatography system (UHPLC, Vanquish, Thermo Fisher Scientific Inc., Waltham, MA, United States) with a column (ACQUITY UPLC HSS T3 Column, 100 Å, 1.8 μm, 2.1 mm × 150 mm, Waters Corporation, United States), column temperature: 40°C, injection volume: 2 μL, flow rate: 0.3 mL/min, mobile phases A: 0.1% formic acid in water, B: 0.1% formic acid in methanol, C: 0.05% acetic acid in water, D: 0.05% acetic acid in methanol. QC samples are interspersed throughout the sampling process to monitor and evaluate the stability of the system and the reliability of the experimental data, and the QC samples are prepared by taking equal amounts of samples and mixing them.

The samples were separated by UHPLC and analyzed by mass spectrometry using a Q Exactive HF-X mass spectrometer (Thermo Fisher Scientific Inc., Waltham, MA, United States), each sample being individually positively and negatively ionized using electrospray ionization (ESI) mode detection. The mass spectrometry conditions were as follows: Sheath gas flow rate: 50, auxiliary gas: 13, spray voltage: 2.5KV (+)/2.5KV (−), S-lens RF: 50, capillary temperature: 325°C, auxiliary gas temperature: 300°C, secondary collision energy (NCE): 30, Top *N* = 10. Scan range: 70–1,050 m/z, scan mode: positive and negative ions separately. Analysis software: Xcalibur version 4.1. The raw data obtained from the mass spectrometry acquisition was subjected to data pre-processing such as peak extraction, peak alignment, peak correction and normalization using the TidyMass package. A three-dimensional data matrix consisting of sample name, peak information (including retention time and molecular weight) and peak area was output. Metabolite structure identification was performed by accurate mass number matching (<25 ppm) and secondary spectral matching, searching the laboratory’s own databases as well as databases such as HMDB, Massbank, KEGG, snyder, mona and others.

The SIMCA-P 14.1 software (Umetrics, Umea, Sweden) was used for pattern recognition, and the data were preprocessed by Pareto scaling and then subjected to multidimensional statistical analyses, including unsupervised principal component analysis (PCA), supervised partial least squares discriminant analysis (PLS-DA) and orthogonal partial least squares discriminant analysis (OPLS-DA), and unidimensional statistical analyses, including Student’s *t*-test and analysis of variance. Volcano plots were generated using R software.

### 2.9 Statistical analysis

GraphPad Prism 5 software (GraphPad Software, Inc., San Diego, CA, United States) was used for statistical analysis and presentation. Measurement data were expressed as mean ± standard deviation (x̄±s). Analysis of variance (ANOVA) was used for multi-sample comparisons, two independent samples *t*-test for between-group comparisons, and LSD test for further analysis of differences between two groups. ANOVA, Tukey, Anosim and Metastats tests were used to compare differences in bacterial flora between groups, and a difference of *p* < 0.05 was considered statistically significant.

## 3 Results

### 3.1 V9 effectively improved the symptoms of DSS-induced colitis model mice

All groups of mice (*n* = 6) were treated for 10 days and general assessment indicators were measured and recorded daily. On the 3rd day after drinking the DSS solution, the mice in groups M and V9 began to have irregular and bloody stools, accompanied by lethargy and hyperactivity, dull fur and decreased appetite.

**Figure 2:**
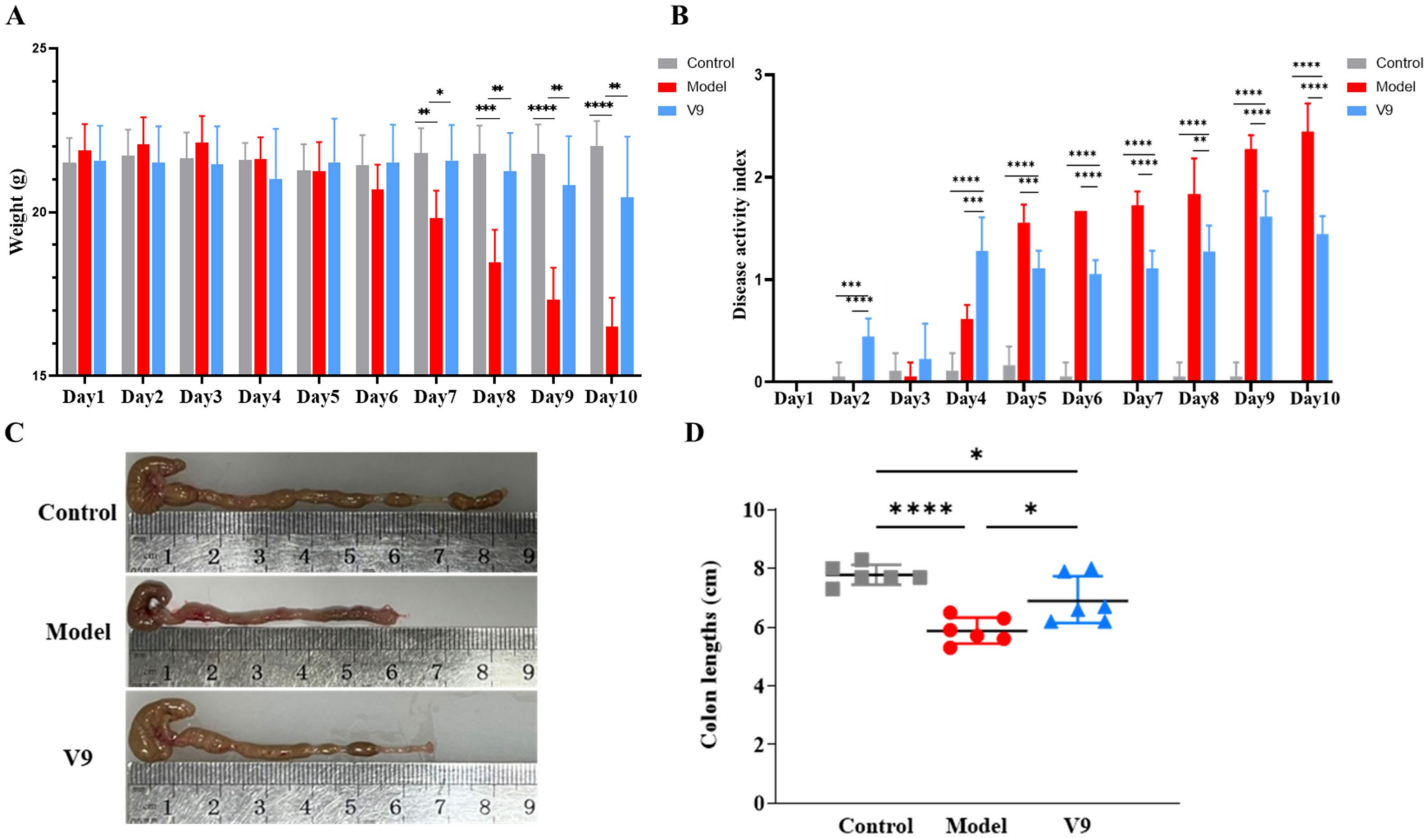
V9 improved body weight, DAI and colon length in mice. **(A)** Plot of changes in body weight of mice in each group. **(B)** Plot of changes in DAI of mice in each group. **(C,D)** Comparison of colon length of mice in each group. *p < 0.05, **p < 0.01, ***p < 0.001, ****p < 0.0001.

The changes in body weight of mice in each group are shown in Figure 2A, where mice in group M showed a continuous decrease in body weight from day 3, with a significant decrease from days 7–10 compared to group C (*p* < 0.01, *p* < 0.001, *p* < 0.0001, *p* < 0.0001, and *p* < 0.0001, respectively). Mice in group V9 started to lose weight slightly on day 4, but there was a slight recovery on days 5–7, and the downward trend slowed down significantly on days 7–10 compared to group M (*p* < 0.05, *p* < 0.01, *p* < 0.01, and *p* < 0.01, respectively). Mice in group C showed little change in body weight throughout the intervention period. The results showed that the intervention with V9 was able to slow down the weight loss of the model mice.

The results of the DAI scores of the mice in each group are shown in Figure 2B. There was no significant change in the DAI scores of mice in group M on days 1–3, which increased sharply from day 4 and peaked on day 10. There were slight fluctuations in the DAI scores of mice in group V9 on days 1–3, and a sharp increase on days 3–4, which slowed down significantly from days 5–10 compared to group M. The DAI scores of mice in group C remained stable throughout the intervention period. On days 4–10 of the V9 intervention, the comparisons between each of the two groups were statistically different (all *p* < 0.01).

At the end of the intervention, the colons of each group of mice were removed and measured for length. The results are shown in Figures 2C,D. Compared to the mice in group C, the colon length of the mice in group M was significantly shorter (*p* < 0.0001), and compared to the mice in group M, the colon length of the mice in group V9 was increased and statistically different (*p* < 0.05).

**Figure 3:**
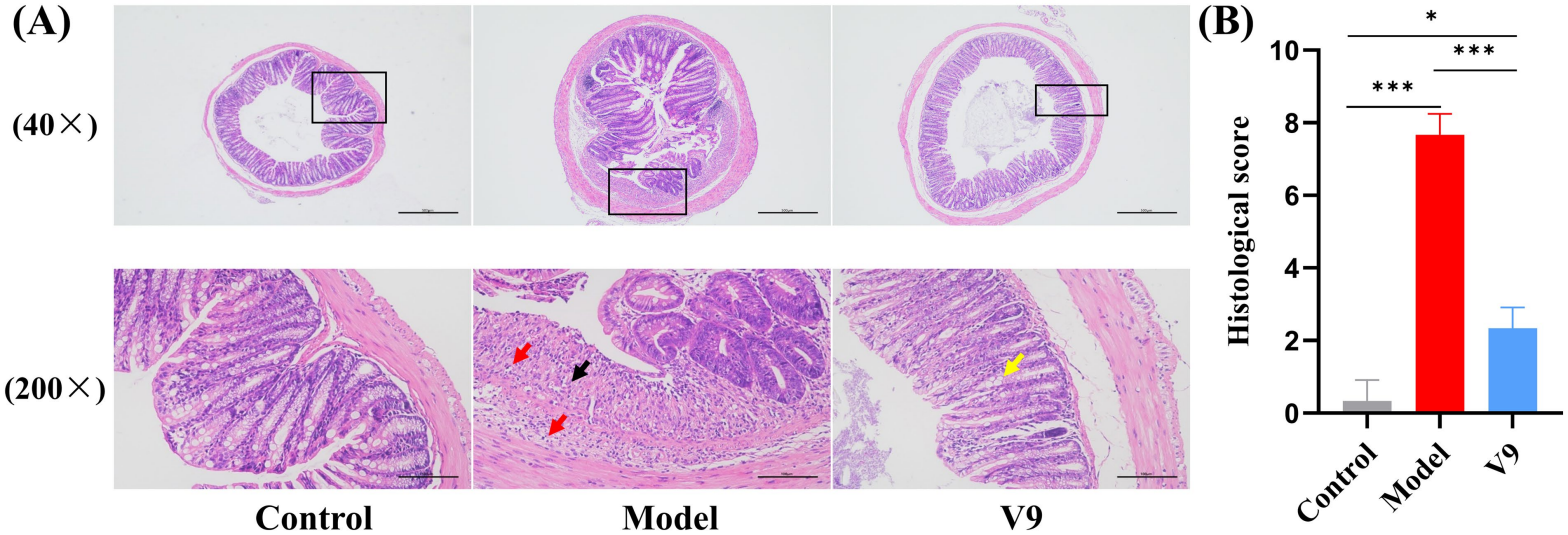
H&E staining of colonic tissues from mice in groups C, M and V9. **(A)** Microscopic images of H&E staining (40× and 200×), the microscopic image (200×) is a magnified image of the area shown in the black box. Black arrows, proliferating connective tissue; red arrows, focal in-filtration of inflammatory cells; yellow arrows, cup cells. Scale bar, 100 μm. **(B)** Histological score. *p < 0.05, **p < 0.01, ***p < 0.001.

At the end of the intervention, colonic tissues from mice in each group were collected for pathological analysis by H&E staining to observe the changes in the morphology of the intestinal mucosa of the colonic tissues (Figure 3). In group M mice, extensive ulceration was observed in the mucosal layer of the colonic tissue, with a reduced number of intestinal glands in the lamina propria, loss of structure and re-placement by hyperplastic connective tissue (black arrows), a small number of intestinal glands with irregular morphology, and a marked reduction in the number of cuprocytes accompanied by a greater infiltration of inflammatory cells (red arrows). Mice in group V9 had an intact mucosal epithelium with neatly arranged epithelial cells, regular morphology of the intestinal glands in the lamina propria and a greater number of cup-shaped cells than in group M (yellow arrows), with no obvious infiltration of inflammatory cells, which was significantly different from group M (*p* < 0.001). The results indicated that the V9 intervention was able to improve the morphology of the intestinal mucosa and, to some extent, the infiltration of inflammatory cells.

### 3.2 Changes in serum levels of related factors

**Figure 4:**
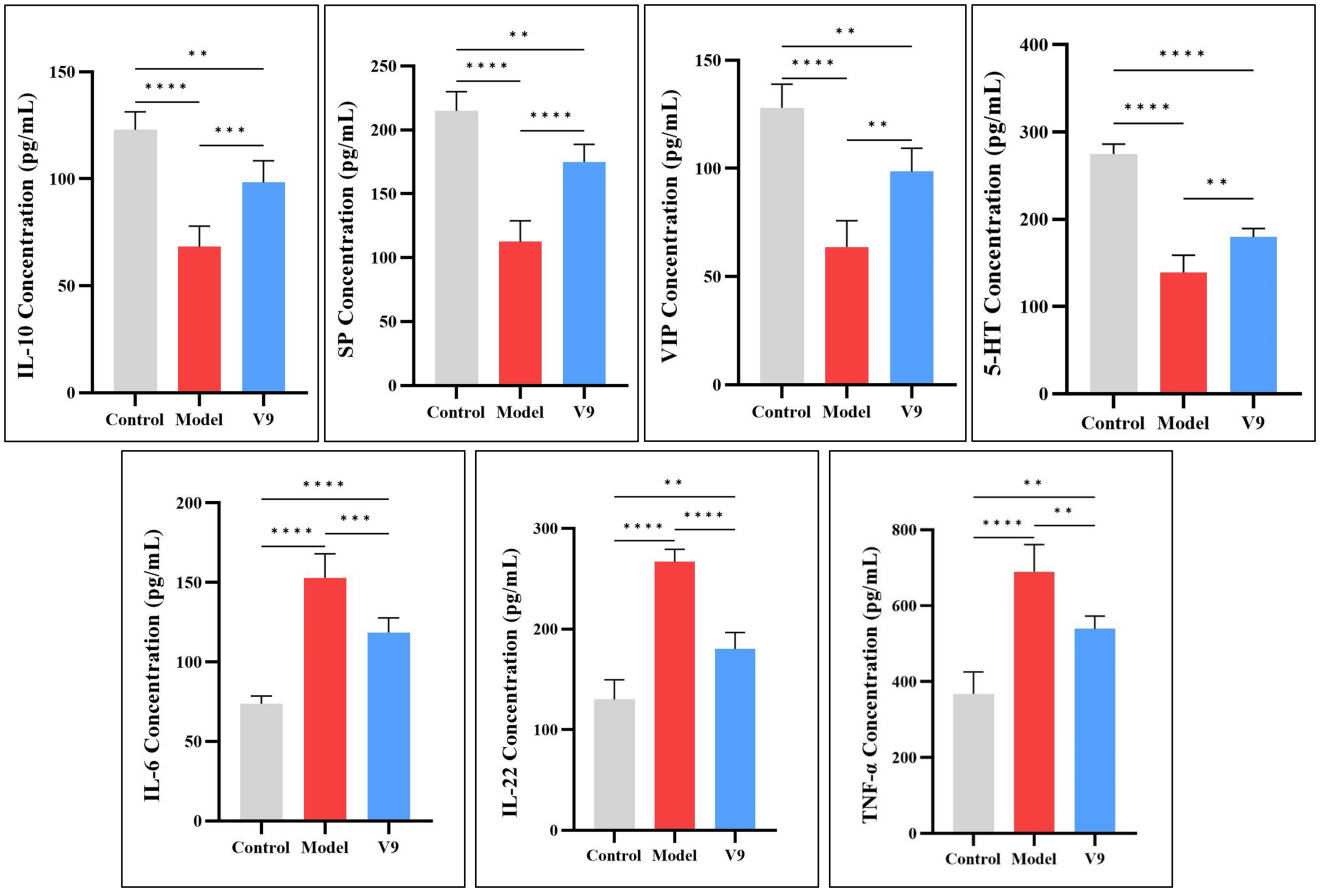
Differences in the expression of IL-10, IL-6, IL-22, TNF-α, SP, VIP, and 5-HT in the serum of mice in groups C, M and V9. *p < 0.05, **p < 0.01, ***p < 0.001, ****p < 0.0001.

After 10 days of intervention in each group of mice, blood supernatants were collected for ELISA to detect the expression of the anti-inflammatory factor IL-10, the pro-inflammatory factors IL-6, IL-22, and TNF-α, and the neurotransmitters SP, VIP, and 5-HT. The results showed (Figure 4) that among the anti-inflammatory factors, the expression of IL-10 was significantly lower (*p* < 0.0001) in mice of group M compared to mice of group C. After V9 intervention, the expression of IL-10 was increased (*p* < 0.001). The trend of serum expression of SP, VIP and 5-HT was similar to that of IL-10, and all of them were statistically significantly different from each other. For pro-inflammatory factors, IL-6 expression was significantly increased in group M mice compared to group C mice (*p* < 0.0001), and IL-6 expression was decreased by V9 intervention (*p* < 0.001). The trend of serum IL-22 and TNF-α expression was similar to that of IL-6, and both were statistically significant.

### 3.3 Changes in the levels of relevant genes in colon tissue

**Figure 5:**
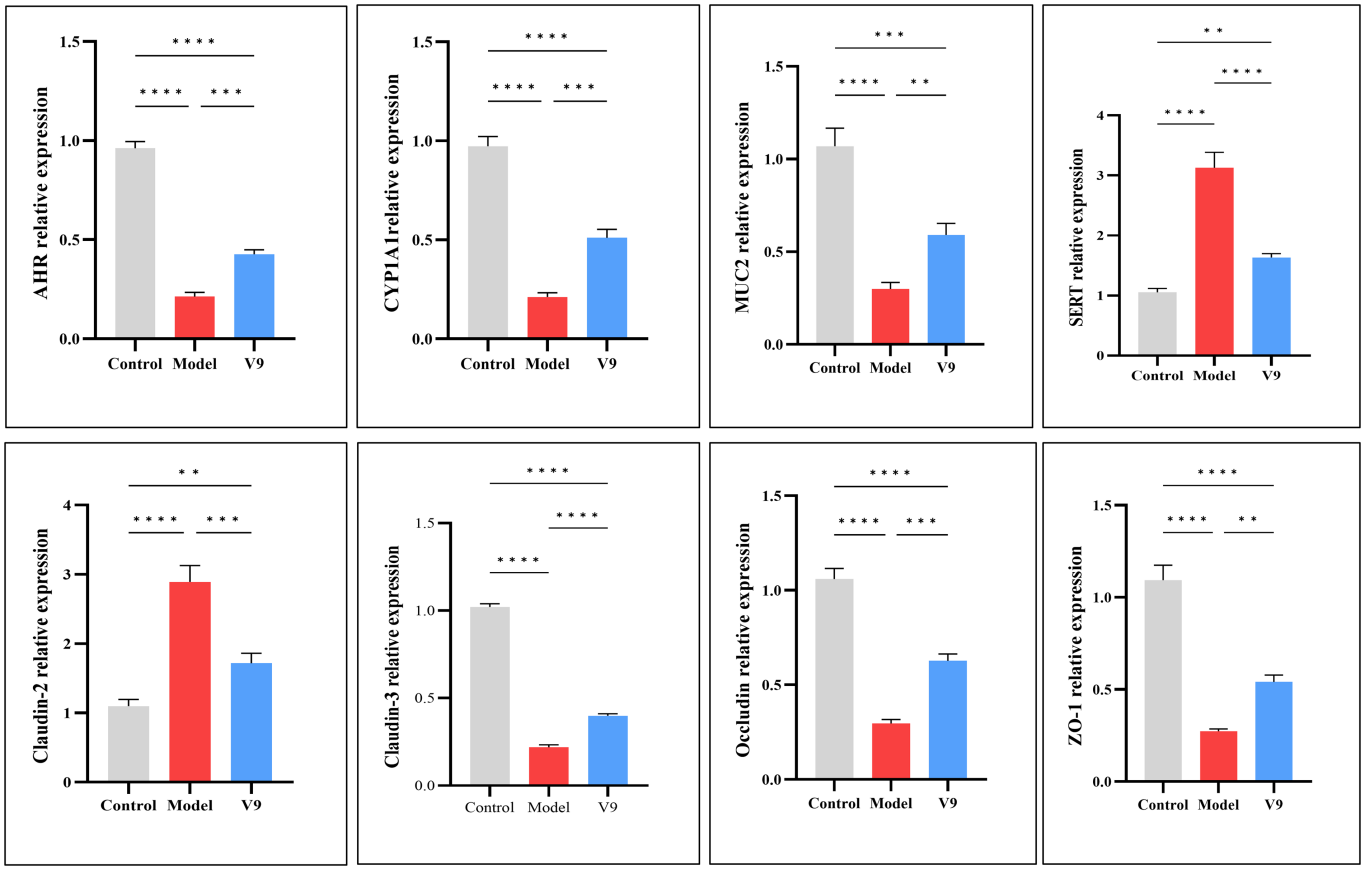
mRNA expression of AHR, CYP1A1, MUC2, SERT, Claudin-2, Claudin-3, Occludin, and ZO-1 in the colonic tissues of mice in groups C, M and V9. *p < 0.05, **p < 0.01, ***p < 0.001, ****p < 0.0001.

After 10 days of intervention in each group of mice, colon tissues were collected to detect the expression of the AHR gene, which maintains the balance of the immune system and regulates the function of the intestinal barrier, and its downstream CYP1A1 gene, the MUC2 gene, which is related to the intestinal barrier and homeostasis, the gene for the transporter protein SERT, and Claudin-2, Claudin-3, Occludin and ZO-1, which encode tight junction proteins. The results of RT-qPCR experiments (Figure 5) showed that the relative mRNA expression of AHR, CYP1A1, MUC2, Claudin-3, Occludin and ZO-1 was significantly decreased in the colonic tissues of mice in group M compared to group C (all *p* < 0.0001). After V9 intervention, the relative mRNA expression of these genes was increased compared to group M (*p* < 0.001, *p* < 0.001, *p* < 0.01, *p* < 0.0001, *p* < 0.001, *p* < 0.01, respectively). In contrast, SERT and Claudin-2 showed opposite results, with increased expression in group M mice compared to group C (both *p* < 0.0001) and decreased relative expression of both genes after V9 intervention, and both were statistically different (*p* < 0.0001, *p* < 0.001, respectively).

### 3.4 Changes in the levels of relevant proteins in colonic tissue

**Figure 6:**
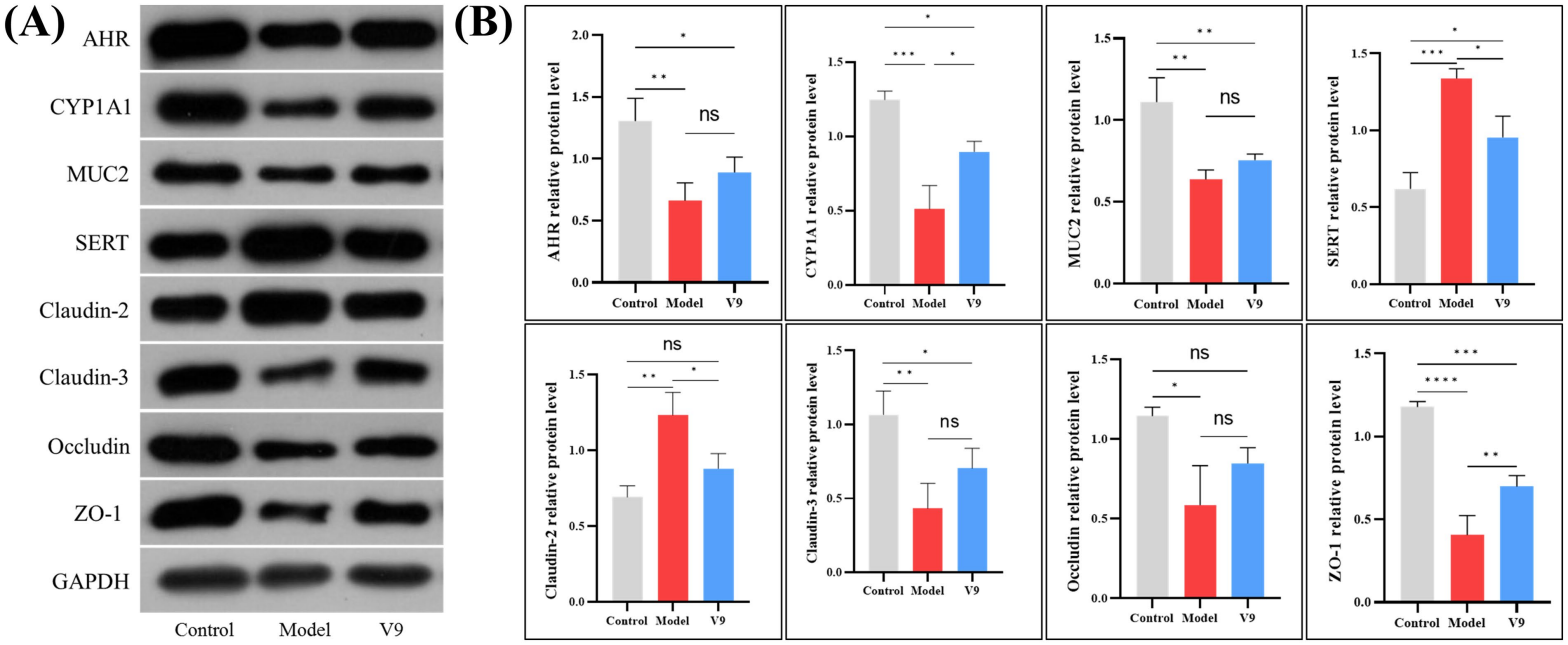
Representative Western blotting **(A)** and densitometric analysis **(B)** of AHR, CYP1A1, MUC2, SERT, Claudin-2, Claudin-3, Occludin, and ZO-1 in colonic tissue from mice of groups C, M and V9. *p < 0.05, **p < 0.01, ***p < 0.001, ****p < 0.0001.

After 10 days of intervention in each group of mice, colon tissue was collected for Western blotting. The experimental results showed (Figure 6) that the relative expression of AHR, CYP1A1, MUC2, Claudin-3, Occludin and ZO-1 was significantly reduced in group M mice compared to group C mice (*p* < 0.01, *p* < 0.001, *p* < 0.01, *p* < 0.01, *p* < 0.01, *p* < 0.05, *p* < 0.0001, respectively). The relative expression of these proteins was increased after V9 intervention (*p* > 0.05, *p* < 0.05, *p* > 0.05, *p* > 0.05, *p* > 0.05, *p* > 0.05, *p* < 0.01, respectively). While the relative expression of SERT and Claudin-2 was significantly higher in group M mice (*p* < 0.001, *p* < 0.01), the relative expression of these two proteins was reduced after V9 intervention and both were statistically different (all *p* < 0.05).

### 3.5 Effects of V9 on gut microbiota

**Figure 7:**
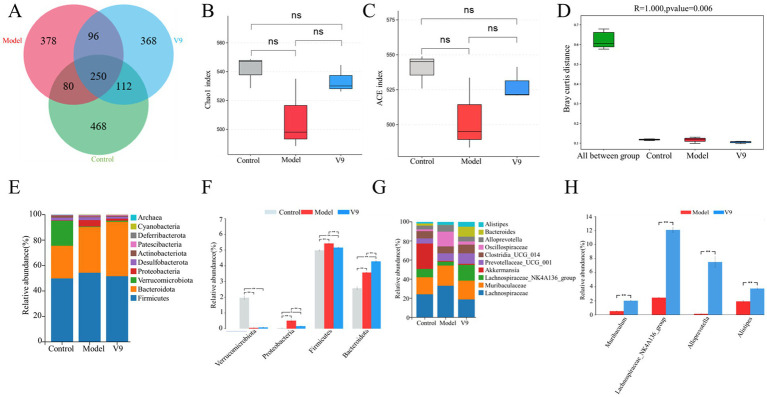
Classification of the gut microbiota of mice in groups C, M and V9. **(A)** Flower plot showing gut microbial OTUs in each group of mice. **(B)** Chao1 index. **(C)** ACE index. **(D)** ANOSIM analysis of gut microbiota in different groups of mice. **(E)** Distribution of gut microbiota at the phyla level. **(F)** Relative abundance of Firmicutes, Bacteroidota, Proteobacteria, and Verrucomi-crobiota at the phyla level. **(G)** Distribution of gut microbiota at the genus level. **(H)** Relative abundance of *Alloprevotella*, *Muribaculum*, *Lachnospiraceae_NK4A136_group* and *Alistipes* at the genus level in groups M and V9. Error lines represent the standard deviation of the mean and were statistically analyzed using Tukey’s multiple comparison test (*p < 0.05; **p < 0.01).

Ten days after the V9 intervention, the microbiota of the cecal contents of the mice was analyzed by 16S rRNA sequencing. The Venn diagram showed a total of 468 OTUs in group C, 378 OTUs in group M, 368 OTUs in group V9 and a total of 250 OTUs in the three groups (Figure 7A). The ACE and Chao1 indices are used to measure species richness, i.e., the number of species. The ACE and Chao1 indices were lower in mice in group M compared with group C, but this difference did not reach statistical significance (both *p* > 0.05), and the V9 intervention attenuated the DSS-induced reduction in species richness (both *p* > 0.05; Figures 7B,C) to a level closer to group C. Significant differences between and within groups were confirmed by the Anosim test (*R* = 1.00, *p* < 0.01; Figure 7D).

We then examined the microbial community structure of each fecal sample. The total fecal microbiota consisted of four major phyla, including Firmicutes, Bacteroidota, Proteobacteria and Verrucomicrobiota (Figure 7E). Compared to group C, group M had significantly more Proteobacteria and Firmicutes (*p* < 0.01 and *p* < 0.01, respectively) and significantly less Verrucomicrobiota (*p* < 0.01; Figure 7F). Compared to group M, group V9 had more Verrucomicrobiota and Bacteroidota (*p* > 0.05 and *p* < 0.01; Figure 7F) and less Proteobacteria and Firmicutes (*p* < 0.01 and *p* < 0.01; Figure 7F). At the genus level (Figure 7G), the top 3 genera detected were *Lachnospiraceae* (13.6–19.7%), *Muribaculaceae* (12.7–14.3%), and *Lachnospiraceae_NK4A136_grou*p (2.4–12.1%). To further analyze the effect of the strains on the gut microbiota of DSS-induced colitis mice, we analyzed the bacterial genera that showed significant changes after the V9 intervention. Significant amounts of *Alloprevotella*, *Muribacu-lum* and *Lachnospiraceae_NK4A136_group*, *Alistipes* were found in the V9 group compared to the M group (all *p* < 0.01; Figure 7H).

### 3.6 Effect of V9 on gut microbiota metabolites

**Figure 8:**
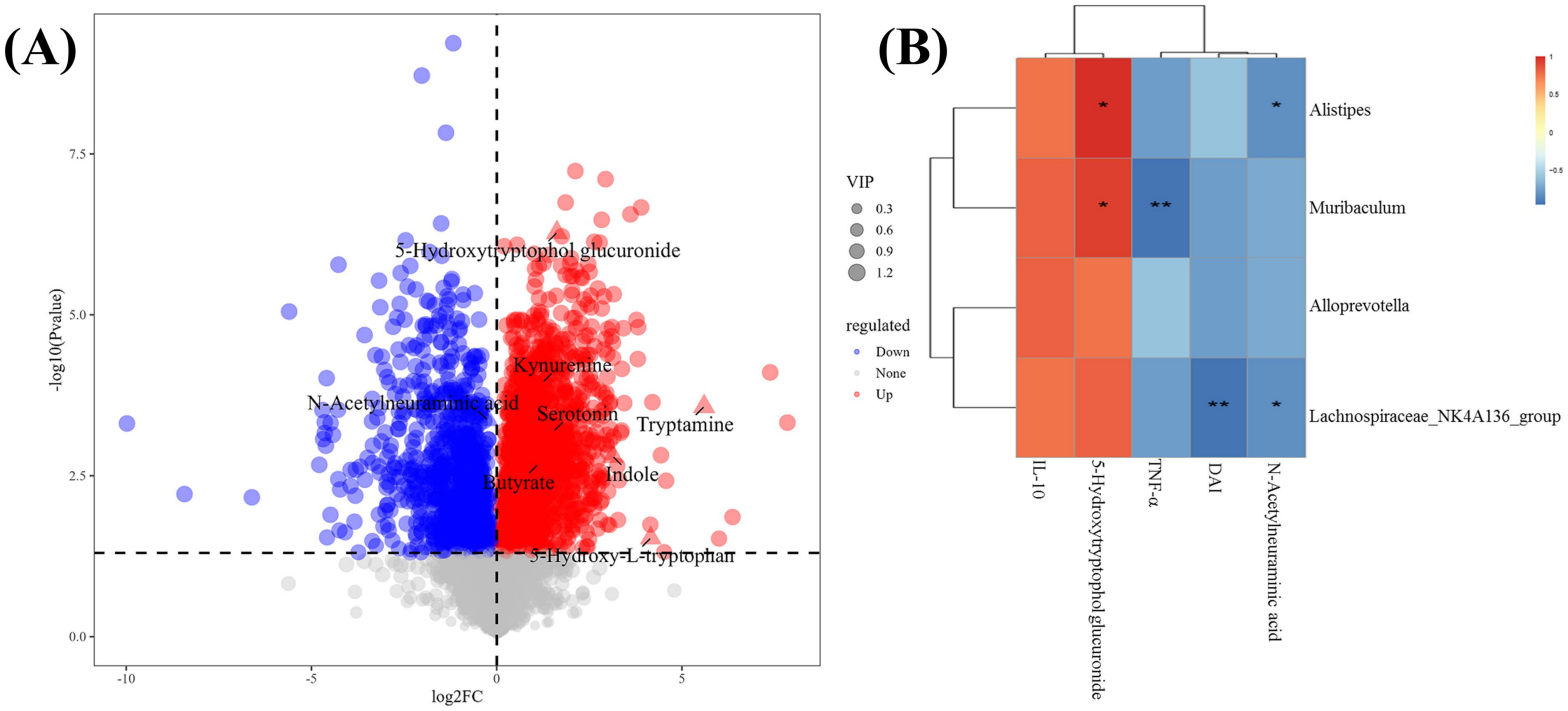
Analysis of differential taxa, metabolites and correlations. **(A)** Volcano plot showing differential metabolites between the gut microbiota of groups M and V9 (*p* < 0.05), each dot rep-resented a detected metabolite and the most important differential metabolites had been annotated. **(B)** Spearman correlation heat map showing the associations between differential taxa, metabolites, serum cytokine levels and disease activity index. * and ** indicate *p* < 0.05 and *p* < 0.01, respectively, and the color scale indicates the *r* value.

The volcano plot shows the *p*-values (*t*-test) and fold change (FC) of the differential metabolites detected in the gut microbiota (Figure 8A). Compared to group M, kynurenine, indole, tryptamine, serotonin, 5-hydroxy-L-tryptophan, 5-hydroxytryptophol-glucuronide (GTOL) and the short-chain fatty acid (butyrate) were upregulated, while N-acetylneuraminic acid was downregulated (*p* < 0.05, Figure 8A).

### 3.7 Correlation between significantly different gut microbiota and gut microbiota metabolites, serum cytokines and DAI

Correlation analysis of cytokines, DAI and differential metabolites with significantly changed general-level bacteria was performed. Several significant associations were found (Figure 8B). *Alistipes*, *Muribaculum* and *Lachnospirace-ae_NK4A136_group* were all positively correlated with 5-hydroxytryptophol glucuronide (*p* < 0.05, *R* = 0.89; *p* < 0.05, *R* = 0.83; *p* > 0.05, *R* = 0.71). *Alistipes*, *Lachnospirace-ae_NK4A136_group* were all inversely correlated with N-acetylneuraminic acid (*p* < 0.05, *R* = −0.83; *p* < 0.05, *R* = −0.83, respectively). In addition, *Muribaculum* was inversely correlated with TNF-α (*p* < 0.01, *R* = −0.94). *Lachnospiraceae_NK4A136_group* was inversely correlated with DAI (*p* < 0.01, *R* = −0.94).

## 4 Discussion

UC is an inflammatory disease of unknown etiology that can severely disrupt patients’ lives and lead to long-term complications. Probiotics, particularly Lactobacillus or Bifidobacterium, may be used as microbiological agents in patients with UC due to their safety, efficacy and ability to restore the intestinal microenvironment (14). In this study, we investigated the beneficial effects of V9 on the clinical manifestations of DSS-induced colitis model mice, where V9 was effective in alleviating UC-associated symptoms with concomitant changes in gut microbiota and metabolites.

DSS is the most commonly used complex to induce UC models in experimental animals and is popular for its ease of use, short and long intervention cycles, and reproducibility (15). In our study, colitis was successfully induced in mice by adding 1.5% (w/v) DSS to the drinking water for 10 days, mimicking UC symptoms. Mice in group M lost weight and had dilute and bloody stools. On day 10, the DAI score of group M was significantly higher than that of group C (Figure 2B), and the colon length of group M was significantly shorter than that of group C and group V9 (Figure 2C). V9 intervention significantly reduced DAI scores, reversed weight loss and alleviated DSS-induced colitis symptoms (Figures 2A–D). Histopathology is the ‘gold standard’ for measuring the severity of UC (16), and V9 was able to significantly attenuate DSS-induced damage to the colon glands, improving the reduction in the number of cuprocytes while reducing the inflammatory cell infiltrate (Figure 3). Taken together, these results supported that V9 treatment was effective in alleviating UC-related symptoms. These results were consistent with those reported in the literature where probiotics had been used to improve colitis (17).

The etiology of UC is complex and it has been suggested that immune system dysfunction and cytokine disruption are the main causes of its pathogenesis (18). Different cytokine levels are strongly associated with the severity of UC. IL-6 is considered to be a key cytokine in the pathogenesis of UC, promoting immune responses and stimulating the production of acute associated reactants (19).

TNF-α is a pleiotropic factor involved in acute and chronic inflammatory and anti-tumor responses, and its inhibitors have been widely used in the treatment of inflammatory bowel disease (20). IL-10 is a recognized anti-inflammatory factor that plays an important role in maintaining tissue homeostasis during infection and inflammation. Studies have shown that excess IL-22 causes neutrophils to overreact and exacerbate barrier damage, leading to colitis (21). In this experiment, IL-22 was significantly increased in the serum of group M mice during DSS-induced colitis, whereas it was significantly reduced after V9 intervention. In conclusion, V9 had a significant anti-inflammatory effect in DSS-treated mice, as evidenced by a decrease in the expression of the pro-inflammatory cytokines IL-6, IL-22 and TNF-α and an increase in the expression of the anti-inflammatory cytokine IL-10 (Figure 4).

Neurotransmitters play a crucial role in regulating neural circuits in the central nervous system and gastrointestinal tract (22, 23). 5-HT is a classic neurotransmitter, 90% of which is synthesized and released by intestinal chromaffin cells (ECs), tryptophan is synthesized into 5-HT by the action of 5-HT synthase (24), and the serotonin trans-porter (SERT) recycles 5-HT to terminate its action. While other studies have shown that 5-HT levels are elevated in models of colitis (25, 26), our results were the opposite, with a decrease in 5-HT levels in group M and instead an increase after the V9 intervention, but it has also been shown that 5-HT attenuates T-cell proliferation and Th1 and Th17 cytokine production *in vitro*. It also reduces the release of IFN-*γ* and IL-17 from CD8+ T cells (27), which are important cytokines in the pathogenesis of inflammatory bowel disease (28, 29). VIP can affect intestinal mucin secretion and intestinal bacterial adhesion by regulating the number and function of intestinal cup cells (30), and a protective effect of VIP against colitis in mice has been reported in the literature (31). SP plays a protective role in maintaining epithelial barrier function (32) and reduces disease severity in colitis mice (33). In our study, V9 was found to increase the serum levels of 5-HT, VIP, and SP in group M mice (Figure 4), and coincidentally, V9 was found to decrease the expression of SERT and reduce the recovery of 5-HT in the serum of colitis mice by Western blotting. V9 was found to promote the recovery of the number of cuprocytes in colitis mice by H&E staining, and promote the secretion of the mucin MUC2 (Figures 5, 6), one of the most prominent components of the intestinal mucus barrier (34). The above results suggest that altering neurotransmitter expression by V9 is another important mechanism for protecting mice from colitis.

The integrity of the intestinal mucosal barrier is essential to protect the intestinal epithelium from invasion by pathogens and is a key factor in the fight against colitis. Tight junction proteins in mechanical barriers are mainly composed of transmembrane proteins (e.g., Claudins, Occludin, etc.) and cytoplasmic proteins (e.g., ZO-1), among others (35). Claudins are important determinants of paracellular permeability. Claudin-3 is a “sealing pore”-like protein whose expression increases the compactness of monolayer epithelial cells, whereas Claudin-2 is a “pore-forming”-like protein whose expression decreases the compactness of epithelial cells and increases solute permeability (36). The results of this study showed that the colonic tissues of group M mice showed a significant increase in the expression of Claudin-2 and a decrease in the expression of Claudin-3, whereas the intervention of V9 improved the expression of Claudin-2 and Claudin-3 in mice with colitis (Figures 5, 6), leading to a more hermetic sealing of the inter-cells and a reduction in the intercellular pore space, which played a protective role against the disruption of the intestinal barrier. Jang et al. (37) showed decreased expression of Occludin and ZO-1 in colitis mice. In this study, V9 increased the expression of Occludin and ZO-1 in the intestinal mucosa of mice with enteritis (Figures 5, 6). All of these results suggest that V9 may alter the expression of tight junction proteins and improve barrier function in mice.

The development of UC is associated with disturbances in the gut microbiota, leading to an overgrowth of harmful bacteria and a decrease in probiotics. This study showed that V9 could mitigate the reduction in species richness induced by DSS (Figures 7B,C), and the Anosim test confirmed that there was a significant difference in species diversity between groups compared to within groups (Figure 7D). These results indicate that V9 administration increased the abundance of gut microbiota in colitis mice. Furthermore, the gut microbiota composition of colitis mice was improved after V9 administration (Figure 7E). Thus, it is possible that microbial species richness and community composition drive the protective effect on UC. Previous studies have shown that the abundance of Proteobacteria and Firmicutes is increased in patients with UC (38), and that high abundance of Proteobacteria is a marker of microbiota dysbiosis (39). The results of the present study showed that at the phylum level, V9 administration reduced the relative abundance of Proteobacteria and Firmicutes in colitis mice (Figure 7F). However, the trend in the relative abundance of Bacteroidota after DSS and V9 interventions was different from previous studies (Figure 7F). This difference may be related to factors such as animal model, coexistence with other pathogenic bacteria and host sex (40) and warrants further investigation.

At the genus level, the relative abundance of *Alloprevotella*, *Muribaculum*, *Lach-nospiraceae_NK4A136_group*, and *Alistipes* was significantly higher in group V9 than in group M (Figure 7H). *Lachnospiraceae_NK4A136_group*, *Alistipes* and *Alloprevotella* are short chain fatty acid (SCFA)-producing bacteria (41–43) with anti-inflammatory capacity in UC mitigation (44). In addition, the results of Spearman correlation analysis in this study showed a significant inverse correlation between *Lachnospiraceae_NK4A136_group* and DAI (Figure 8B). *Alistipes* are also typical tryptophan metabolizing bacteria (45), which can promote an increase in tryptophan metabolites (e.g., kynurenine, indole, etc.), and tryptophan metabolism is not only in-volved in immune tolerance, but is also necessary to maintain microbiota diversity (46, 47). *Muribaculum* is a major mucin monosaccharide forager that may impede Clostridioides difficile colonization, and V9 intervention significantly increased the relative abundance of *Muribaculum*, which was inversely correlated with TNF-α (*p* < 0.01, Figure 8B), and also impaired pathogen colonization and suppressed intestinal inflammation while maintaining intestinal homeostasis (48).

We screened a selection of relevant metabolites by metabolomic analysis of the cecal contents of each group of mice and found that V9 upregulated beneficial metabolites such as the tryptophan metabolite kynurenine, indole, serotonin (49), tryptamine (50), 5-hydroxy-L-tryptophan (51), 5-hydroxytryptophol glucuronide (52), and the short-chain fatty acid butyrate, and could downregulate the harmful metabolite N-acetylneuraminic acid (Figure 8A). Tryptophan metabolites are strongly associated with the pathogenesis of UC (53) and their reduced levels are also associated with impaired epithelial barrier in UC (54). Intestinal flora can directly metabolize tryptophan to indole, serotonin and other metabolites. These metabolites can bind to AHR and trigger various post-immune transcriptional responses (55), thereby maintaining immune homeostasis and intestinal barrier function (49). Many AHR ligands are degraded and inactivated by cytochrome P450 family proteins (e.g., CYP1A1), which are transcriptional targets that act directly on the AHR and form a feedback loop in the AHR signaling pathway (56). In this experiment, the expression of AHR in the intestinal mucosa of mice in group V9 was significantly higher than that in group M (Figures 5, 6). To verify the active effect of AHR, this experiment examined the mRNA and protein expression of its downstream effector molecule, CYP1A1 (57), and found that V9 intervention could significantly increase the expression of CYP1A1, both consistent with the expression of AHR (Figures 5, 6), which further demonstrated that V9 could in-crease intestinal tryptophan metabolites. These tryptophan metabolites could act as ligands to activate the AHR, which in turn maintained immune homeostasis and intestinal barrier function, and regulated the role of its downstream effector molecule CYP1A1 to ensure the dynamic balance of AHR signaling (56). Among the SCFAs, butyrate is one of the best studied and plays a key role in colonic epithelial homeostasis (58). Butyrate has been used experimentally in the treatment of colitis with favorable results (59). N-acetylneuraminic acid plays a role in immune evasion in *Campylobacter jejuni* (60), neuroinvasive *Escherichia coli* K1 (61). Pathogen use of N-acetylneuraminic acid as a pathway for invasion and colonization may exacerbate infectious enteritis. In this study, we found that *Alistipes*, *Lachnospiraceae_NK4A136_group* were all inversely correlated with N-acetylneuraminic acid (Figure 8B), suggesting that V9 promoted the growth of beneficial bacteria, up-regulated the beneficial metabolites, inhibited the colonization of harmful bacteria and down-regulated the harmful metabolites, which in turn alleviates enteritis.

## 5 Conclusion

V9 could alleviate the symptoms of DSS-induced colitis in mice. In the future, it is necessary to further explore the specific mechanisms of V9 to alleviate colitis, such as the specific mechanisms between V9 and tryptophan metabolism, 5-hydroxytryptamine and AHR, and to continue relevant longitudinal studies to pro-vide more evidence for the application of V9 in the treatment and prevention of UC.

## Data Availability

The original contributions presented in the study are included in the article/supplementary material, further inquiries can be directed to the corresponding authors.
